# Volume Determination Challenges in Waste Sorting Facilities: Observations and Strategies

**DOI:** 10.3390/s24072114

**Published:** 2024-03-26

**Authors:** Tom Maus, Nico Zengeler, Dorothee Sänger, Tobias Glasmachers

**Affiliations:** 1 Institut für Neuroinformatik, Ruhr-Universität Bochum, 44780 Bochum, Germany; nico.zengeler@ini.rub.de (N.Z.); tobias.glasmachers@ini.rub.de (T.G.); 2 Sutco RecyclingTechnik GmbH, Britanniahütte 14, 51469 Bergisch Gladbach, Germany; dorothee.saenger@sutco.de

**Keywords:** waste sorting facilities, volume determination, ultrasonic sensors, machine learning

## Abstract

In this case study on volume determination in waste sorting facilities, we evaluate the effectiveness of ultrasonic sensors and address waste-material-specific challenges. Although ultrasonic sensors offer a cost-effective automation solution, their accuracy is affected by irregular waste shapes, varied compositions, and environmental factors. Notable inconsistencies in volume measurements between storage bunkers and conveyor belts underscore the need for a comprehensive approach to standardize bale production. With prediction reliability being constrained by limited datasets, undocumented modifications to machine settings, and sensor failures, this task renders a challenging application area for machine learning. We explore related research and present dataset analyses from three distinct waste sorting facilities in Europe, addressing issues such as sensor usability, data quality, and material specifics. Our analysis suggests promising strategies and future directions for enhancing waste volume measurement accuracy, ultimately aiming to advance sustainable waste management.

## 1. Introduction

Waste management, a crucial pillar of our global environmental infrastructure, has undergone remarkable evolution and enhancement over time [1,2]. One area commanding attention focuses on the optimization of waste sorting facilities. Various emerging technologies, like robotics, sensor technology, and AI-driven optimization, have begun to contribute to the automation and streamlining of sorting processes [1,3].

In waste sorting facilities, materials are separated and typically stored in dedicated bunkers. These bunkers are connected via conveyor belts to mechanical presses, which compress the sorted materials into uniform bales. These compressed bales become valuable raw materials for various industries, fostering a circular economy by supporting the production of new goods. Standardizing their size turns out crucial for efficient storage and transportation logistics. A critical aspect of waste sorting facilities revolves around accurately monitoring and quantifying the volume of waste within the storage compartments after the sorting process is completed.

**Figure 1:**
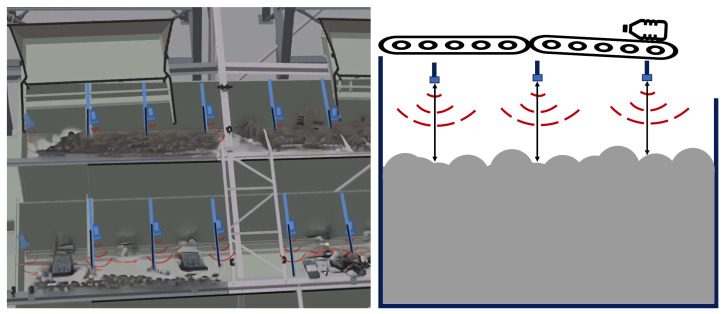
An exemplary illustration of the discussed setting. On the **left**, two bunkers with rows of multiple ultrasonic sensors are shown from above. On the **right**, a cross-section through one bunker demonstrates how multiple sensors scan the distance to the waste piles to estimate the volume.

Using ultrasonic sensors for distance measurement as a precursor to volume determination offers a cost-effective alternative to employing large-scale weighing, being hardly practicable in dynamic systems with multiple large bunkers [4,5]. These sensors function by emitting ultrasonic waves and measuring the time taken for the emitted wave to rebound after hitting the object’s surface [6]. Given the known speed of sound in the air, the calculation of distance to the object becomes possible. In a more complex setup, using multiple sensors in a row-like arrangement allows for a rudimentary representation of the waste pile, thus providing an estimate of its volume (see Figure 1).

Despite the utility of ultrasonic sensors, they introduce a unique set of challenges. Various factors such as (1) the irregular shapes of waste piles, (2) variability in waste material composition affecting the propagation of ultrasonic waves, (3) differing densities of waste material, and (4) inherent or external noise all can impact the accuracy of volume measurements [7,8]. Waste piles within bunkers are inherently dynamic, constantly evolving in both size and shape due to ongoing waste inflow, shifting, and removal. This dynamic nature poses significant challenges when attempting to precisely and consistently measure the volume using point-distance measurements. The distance measurements only give limited information about the total volume distribution. When motion occurs inside the bunker, for example during pile shifting under the input area or emptying, it may lead to temporarily unreliable volume estimates. Regarding the effect of inherent noise on sensors, it has been stated that even distinct sensors of the same model may show unique qualities [7]. Further, functioning within a dynamic and noisy environment filled with auditory disturbances can substantially distort the sensor readings. These disturbances could stem from the roar of heavy machinery to the impact noise of waste material dumped into the bunker, or even changes in temperature and humidity [8]. The volume estimates are calculated by an algorithm, taking into account all current sensor readings from the row per bunker. To mitigate the negative effect of outlier values caused by sensor malfunctions, the use of advanced signal processing techniques becomes necessary, aiming to filter out noise and outliers to extract true distance measurements [7,8,9]. Furthermore, material-specific characteristics of the consistency between input and output volume are examined.

In this paper, we delve into specific challenges associated with improving efficiency and accuracy in waste volume determination and handling. We present related research in this field, typical data structures relevant to this context, specific challenges, and potential solutions, while outlining future directions for the field. Our research identifies a critical need for better measurement techniques amidst noise and material variability, while also showing that accurate bale length estimation remains elusive, despite precise volume and weight data. We investigate in detail whether and to what extent the shortcomings of ultrasonic sensors can be compensated by elaborate data post-processing using machine learning methods.

Our work thus underscores the need for ongoing development in waste sorting technologies to overcome these limitations.

## 2. Related Research

Research in sensor technology and machine learning algorithms lays the groundwork for diverse applications, including waste management. This section reviews key literature with a connection to waste sorting in three areas: sensor usage, machine learning algorithms, and computer vision techniques.

### 2.1. Usage of Sensors

To obtain an estimate of the volume, distance measurements can be utilized, commonly implemented through the use of low-cost ultrasonic (US) and optical sensors [10]. Ultrasonic sensors emit sound waves to precisely measure distances, while laser sensors rely on light and the reflection principle for distance calculation. Studies have demonstrated that laser sensors are more accurate and exhibit lower error rates in distance measurements across various surface types [11,12]. During comparative testing of both optical and ultrasonic sensors, each sensor type manifested distinctive accuracy profiles contingent upon specific material under scrutiny [10]. Numerous research efforts address the advancement of IoT (“Internet of Things”)-enabled smart waste management systems, aiming to address the issue of overflowing garbage bins and its associated environmental pollution [13]. This research involves augmenting traditional waste containers with a combination of ultrasonic, laser, and weight sensors, facilitating the real-time monitoring of waste levels [14]. Thakker et al. [2] described a system named “Smart Garbage Bin”, which notifies when it nears full capacity, facilitating timely waste collection and mitigating garbage overflow, thus addressing environmental and health concerns. Additionally, the authors propose a method employing Near-Infrared (NIR) spectroscopy to differentiate between five types of non-biodegradable plastic resins. This separation process allows for the extraction of biodegradable waste, which can subsequently be utilized for biogas production [2].

Another proposed system by Aguila et al. incorporates ultrasonic sensors for volume measurement, load cells for weight monitoring, and an Arduino Uno microcontroller for system control. The researchers in this study further optimized the placement of ultrasonic sensors to achieve the highest data accuracy [14].

### 2.2. Usage of Algorithms and Machine Learning

Characterizing the volume of bulky materials commonly found in waste poses a challenging task addressed by multiple approaches, including formulating mathematical models that describe the volume and shape of large granular piles of bulk materials in bunkers [15].

The exploration of machine learning applications currently emerges as another relevant theme in the literature, offering alternative innovative approaches to traditional solid waste management built upon mechanistic models and rigid algorithms. These conventional approaches often fall short, especially when dealing with limited data. In such situations, AI-powered models may yield more promising and effective solutions [16].

Rutqvist et al. [1] offer a compelling instance of this approach. In their work, the authors successfully utilize automated machine learning to detect when recycling containers are emptied, based solely on sensor readings. This implementation significantly improves accuracy and recall rates. Their highest-performing solution leverages a Random Forest classifier, resulting in enhanced predictions for the emptying times of recycling containers.

Bae et al. [9] introduce a unique data processing algorithm for the smoothing and outlier removal of water level data gathered by ultrasonic sensors. This approach effectively addresses the issue of random errors, such as those caused by water waves, often found in data collected from dynamic environments like waste management sites. The introduced algorithm demonstrates promising outcomes, effectively handling outliers and substantial random error, which contributes to improved accuracy and usability.

Sahoo et al. [8] tackle the challenge of enhancing sensor measurement precision. Their novel approach employs a modified neural network architecture, the Levenberg–Marquardt backpropagation artificial neural network (LMBP-ANN), to mitigate the impacts of environmental conditions and noise on distance measurements made with low-cost ultrasonic sensors. The proposed model reduces measurement error to within ±1 cm for distances ranging from 2 cm to 500 cm and extends the maximum standard operating range of the ultrasonic sensor from 400 cm to 500 cm. This significant enhancement in measurement accuracy and range demonstrates its applicability in contexts where manual and contact-based distance measurements remain challenging.

In the industrial context, Bowler et al. [7] stress the importance of ultrasonic sensors in optimizing manufacturing processes during the fourth industrial revolution. Using supervised machine learning models, they aim to connect ultrasonic sensor data with valuable manufacturing insights. However, this requires labeled data, which can often be challenging to obtain in factory settings. To address this, they explore two domain adaptation methods that enable model transfer without labeled data. Their research demonstrates the effectiveness of these methods in monitoring industrial processes, highlighting the potential of combining ultrasonic measurements with transfer learning techniques.

### 2.3. Usage of Computer Vision and Related Methods

An alternative to distance measurements includes the utilization of stereo camera vision systems coupled with computer vision. The latter has attracted growing attention in the realm of waste sorting, capitalizing on advancements in computational power and algorithms [17].

One noteworthy application of computer vision is its combination with hierarchical deep learning to enhance waste detection and classification [18]. However, a possible limitation of CV lies in differentiating materials that share similar appearances [17].

Stereo cameras may, in turn, exhibit suboptimal performance under specific conditions such as “plain walls, glass surfaces, or poor lighting” [19]. Integrating optical and ultrasonic sensors could therefore enhance the overall capabilities of the vision system.

Other cutting-edge technologies, such as LIDAR (“Light Detecting and Ranging”) and 3D Laser scanners, are currently under development by various companies. For example, LIDAR scanners have been employed to monitor the vegetative growth in strawberry plants, including volume estimates from 3D point clouds [20]. These technologies may be applied to accurately determine the volume of different materials within industrial bunkers and conveyor belts. However, it is worth noting that these new scanners are significantly more expensive than US sensors, preventing them from being a comprehensive alternative for many applications.

In sum, these studies spotlight strategies and techniques that harness sensor technology in optimizing volume determination for industrial processes. The overlapping themes of machine learning applications, advanced data processing techniques, and improvements to sensor-based measurement accuracy signal the potential for substantial innovation in this field. Future research can further fine-tune these methods for specific applications and contexts, building upon the ideas and discoveries showcased in these studies.

## 3. US-based Volume Determination

To understand the challenges associated with volume determination based on measurements from ultrasonic sensors, one must first delve into the underlying available data and its characteristics. The data repository analyzed in this study encompassed details from three distinct European facilities. This data pertained to components such as bunkers, conveyor belts, and presses and included categories like sensor measurements, activity status, energy consumption, and calculated volumes, each paired with a timestamp (refer to Appendix A, Table A1 and Table A2 for exemplary categories).

In those waste management facilities, multiple bunker dimensions (e.g., 10 m × 2 m × 1.8 m) and filling positions (left, middle, right), which determine the entry point of the sorted waste into the bunker, were observed. To monitor these bunkers effectively, multiple ultrasonic (US) sensors of the model microsonic mic + 340 were strategically installed. These devices utilize the principle of echo propagation time measurement to determine distances, featuring an operational range of up to 3400 mm. The sensors demonstrate a resolution between 0.18 mm and 1.5 mm and operate at a transducer frequency of 120 kHz [21]. They were positioned centrally above each bunker in regular intervals at a height of approximately 200 cm. This arrangement ensured minimal interference between sensors, optimizing their performance for accurate data collection. The number of sensors per bunker varied, depending on the size of the bunker, with an average allocation of approximately ten sensors per bunker.

The data structure was largely constituted of metrics related to waste material processing. A critical aspect was the calculation of bunker volume, derived from multiple readings from ultrasonic sensors. An algorithm designed to manage partial sensor outages was used to estimate volume based on sensor readings, thus enhancing estimates’ accuracy and reliability (see Appendix A, Algorithm A1). To calculate the average material height, it integrates valid sensor readings and, in case of an invalid reading, the algorithm deduces plausible values using data from neighboring sensors. After calculating the mean height, multiplication by the bunker’s width and length yields the volume estimate. Ensuring accurate volume estimates is pivotal. It helps in determining the optimal time to empty the bunker, guaranteeing that both the conveyor belt and press are available and that an integer number of bales can be produced.

We were particularly interested in three specific relationships: bunker-to-belt volume, bunker-to-bale volume, and belt-to-bale volume. These relationships are defined as follows:

$$F_{1}=\frac{V_{\mathrm{belt}}}{V_{\mathrm{bunker}}}\;F_{2}=\frac{V_{\mathrm{belt}}}{V_{\mathrm{bale}}}\;F_{3}=\frac{V_{\mathrm{bunker}}}{V_{\mathrm{bale}}}$$

Observations indicate that the volume measured on the belt using lasers tends to be a more accurate predictor for operating the press than the volume measured in the bunker. However, this belt volume data is available only after the bunker volume has already initiated the emptying process. For earlier estimates of $V_{\mathrm{belt}}$ , we can derive the factor $F_{1}$ for each bunker and material type from previous emptying events. This prediction method relies on the median of the last 10 values of $F_{1}$ recorded for the same material.

The volume inside the bunker ( $V_{\mathrm{bunker}}$ ) is notably larger than that of a bale ( $V_{\mathrm{bale}}$ ). The pressing process compacts the loose material from the bunker, effectively removing air. For example, 10 m^3^ of material in the bunker might compress down to a 1.4 m^3^ bale, or in some cases like films, 30 m^3^ may be compressed to the same bale volume. After the pressing process, measurements were taken to determine both the length and weight of the current bale. The focus of this study was to address the raw sensory data from bunkers filled with various waste materials (see Appendix A, Table A3).

The datasets examined in this study did not contain missing values, except specific periods during which the automatic bunker management system was deactivated and hence no data was logged. However, it is important to acknowledge that several sensors exhibited extreme unreliability, generating substantial outliers. These outliers should be treated as missing values. This adds complexity to the analysis as these implausible values must be identified. Timestamps were consistently maintained, enhancing the temporal coherence of the dataset. The data ranged from 2022 to 2023, with each dataset encompassing approximately 4 to 6 months. Nevertheless, certain metrics, especially energy data, remained unrecorded in the dataset used for our analysis. Moreover, distinct recorded values among all material types exhibited significant deviations from the anticipated ranges.

## 4. Challenges

Determining volume in waste sorting facilities is faced with numerous challenges, each requiring in-depth analysis and distinct solutions.

### 4.1. Volume Ratio Is Highly Unstable

**Figure 2:**
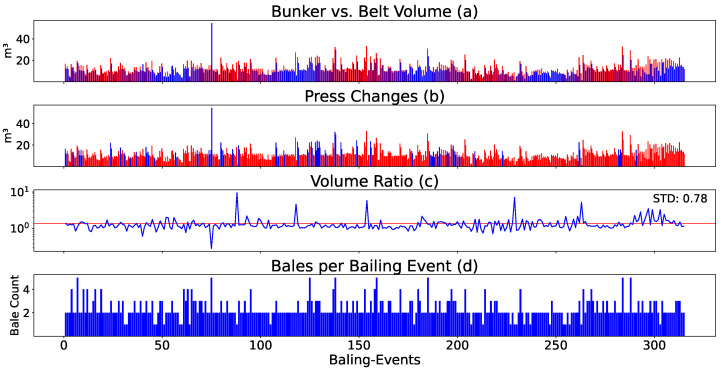
This figure offers an analysis of volume flows in a waste sorting facility with two presses. It juxtaposes the volume measured in the bunker (red) and belt (blue) (**a**), shows the selection of press 1 (blue) or press 2 (red) based on baling event frequency (**b**), presents the variation (trimmed) in the bunker/belt ratio around its mean (**c**), and quantifies bales pressed per baling event (**d**).

Insights into the volume flow dynamics are given by a specific bunker storing PET from a waste sorting facility with two presses over approximately six months (a few hundred emptying cycles), shown in Figure 2. We observed distinct differences between the volume measured inside the storage bunker using ultrasonic sensor technology, and the volume measured on the conveyor belt with laser sensors, of which the latter mostly measured lower volumes (see Figure 2a). The figure further illustrates the frequency of press changes, revealing that one press consistently exhibited higher usage than the other. However, this pattern was not uniform across all bunkers (see Figure 2b). We could observe fluctuations around the average ratio of volume between the bunker and belt. Pronounced spikes in the data were observed (see Figure 2c).

The number of bales produced during each baling event typically ranged between 2 and 4 (see Figure 2d).

### 4.2. US Sensors Show Serious Fluctuation

**Figure 3:**
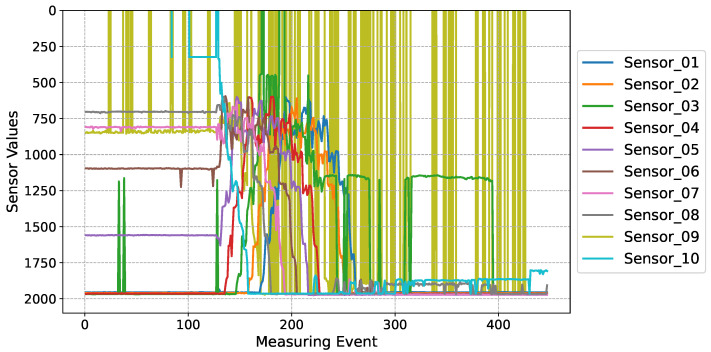
Graphical representation of raw sensor outputs from a designated bunker during the emptying phase, captured over intervals of 1–2 s. A discernible malfunction is evident in e.g., Sensor_03 and Sensor_09.

**Figure 4:**
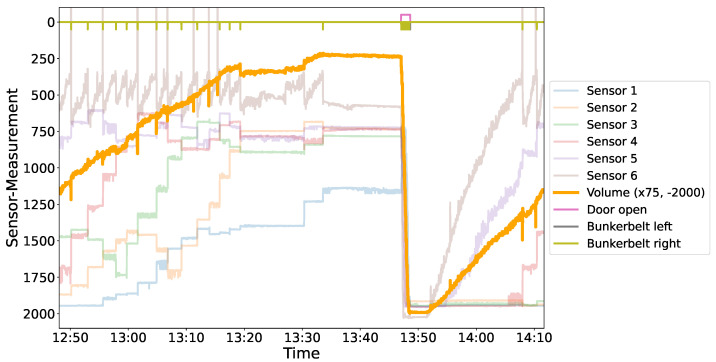
The Figure presents a detailed analysis of sensor readings obtained from a specific bunker (“PP_nat”). The bold orange line represents the trend of volume, calculated using Algorithm A1 (see Appendix A) and scaled for better visibility as explained in the the legend. Opening of the bunker doors marks the specific time designated for bunker emptying, highlighted at the top of the figure. The movement of the belt inside the bunker is further displayed, aiding in understanding the shifting distance measurements. Notably, Sensor 6 (brown) exhibits values outside the plausible range (0–2036 mm). The visual representation utilizes an inverted y-axis for clarity, indicating that at the bottom, the bunker is empty and sensors measure down to the ground of the bunker while decreasing values indicate the filling of the bunker (opaque lines).

**Figure 5:**
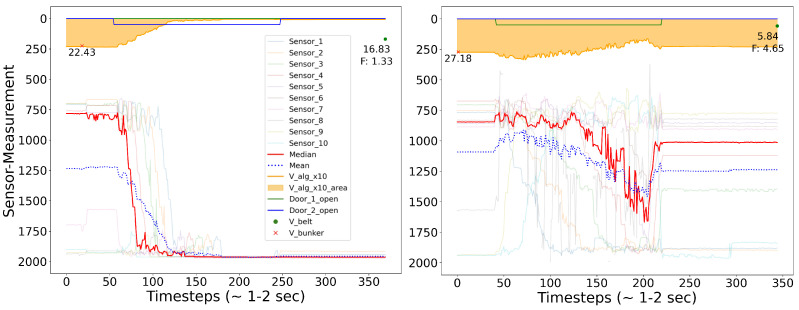
These graphs illustrate both a successful (upper) and unsuccessful (lower) emptying process. It shows that once the computed volume exceeds a certain threshold (red cross), a door opens (indicated by a shifted line at the top) and the bunker empties. The raw sensor values (translucent, in mm), their mean (red), and median (blue) are compared to the volume calculated by the algorithm (see Appendix A, Algorithm A1), magnified by factor 10× for visual purposes (yellow area and curve, in m^3^). The measurement from the laser sensors on the conveyor belt and the resulting ratio between this value and the previously measured Bunker Volume is shown as F.

A primary challenge involves precisely gauging the bunker volume to determine the optimal time of withdrawal. Figure 3, Figure 4 and Figure 5 augment our grasp of the problems related to bunker occupancy by offering a detailed picture of sensor readings. The continuous filling of the bunker with regular shifting of the material inside the bunker by moving the bunker belt can be seen in Figure 3, also showing the spikes of a particular sensor during such movements inside the bunker. Frequent values above or below the given range by certain sensors were regularly observed, as e.g., seen for Sensor 9 in Figure 4. For some materials, multiple sensor malfunctions were observed in parallel. The variance in reading quality increases the complexity and unpredictability of sensor measurements, complicating the task of maintaining consistency.

Figure 5 depicts the sensor behavior during the bunker emptying process. When the bunker volume exceeded a certain threshold, a door opened for emptying. However, even after the emptying process, some cases were observed (lower plot in Figure 5) where sensors still indicated a large amount of material remaining inside the bunker. It is important to note that material is continuously added to the bunker, even during the emptying process, thus preventing it from becoming empty. These malfunctions may play a crucial role in understanding deviations in the volume ratio.

Another challenge arose when emptying bunkers with two presses and corresponding doors. By continuously adding additional waste into the bunker, when opening the door closer to the waste entry point, new waste was automatically added on top of the volume being transported to the press. However, opening the opposite door could lead to trailing waste crowding the bunker’s bottom, resulting in a reduced maximum filling level.

### 4.3. Effect of Multiple Factors (Material, Bale Number)

**Figure 6:**
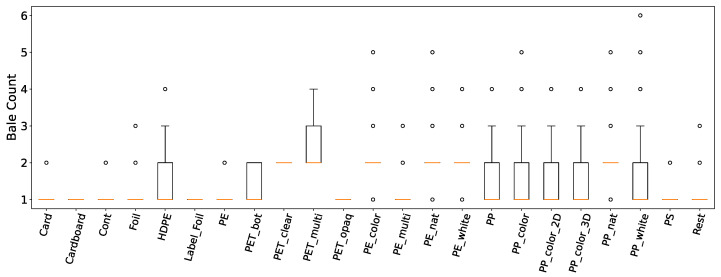
Boxplots displaying the distribution of the number of bales produced per emptying cycle per material in a selected waste sorting facility over a period of approximately 5 months.

Multiple factors, including the number of bales pressed per baling cycle and the material type, are important for understanding volume ratios. The distribution of the number of bales produced per emptying cycle per material is illustrated in Figure 6. Despite some materials having a limited number of data points, a consistent trend emerged, showing that most withdrawals result in an average of 1 to 2 compressed bales.

**Figure 7:**
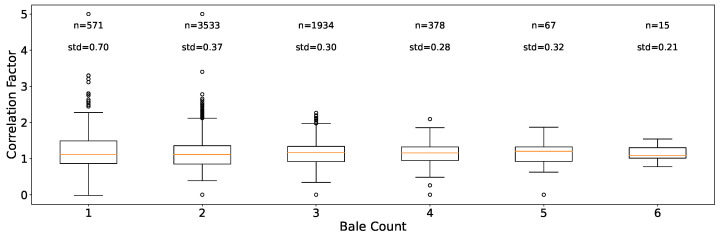
Boxplots illustrating the volume ratio F as a function of the number of bales pressed at a selected waste sorting facility. They display the distribution of data points, while the number of samples and the standard deviation are provided for additional context.

The relationship between the volume ratio and the number of bales pressed at a waste sorting facility is presented in Figure 7. We observed a tendency in the standard deviation to decrease as the number of bales pressed increased.

This finding gains significance when comparing it with information from Figure 6, which reveals that often lower bale numbers (e.g., 1 or 2) are pressed, resulting in greater variability in the volume ratio.

**Figure 8:**
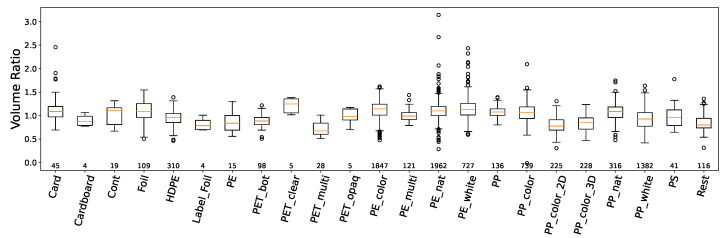
Volume ratio variation (belt volume/bunker volume) per material type (varying sample sizes) at a waste sorting facility over 5 months. The numbers indicate the available data points per material (n).

In Figure 8, we observe the volume ratio in relation to material type at our waste sorting facility. Certain materials exhibited more significant variations in their volume ratios, which may be attributed to physical properties unique to each material. Distinct groups, like PE, exhibit similar tendencies.

### 4.4. Previous Material Influences Current Pressing

**Figure 9:**
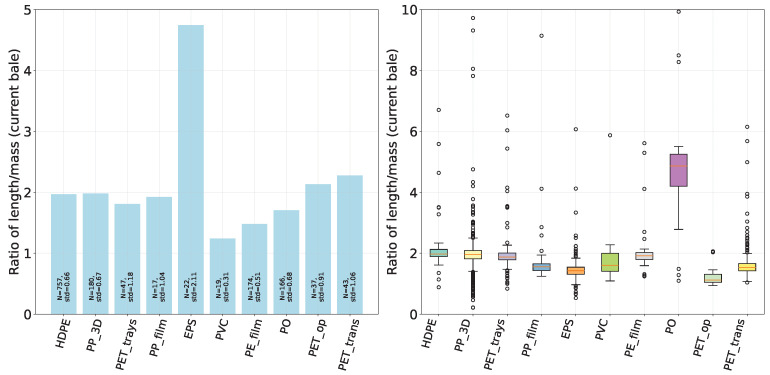
Influence of previous bale material on length-to-mass ratio of newly pressed HDPE Bale. Mean ratios are shown on the left, while the right side displays a boxplot representation, including minimum, maximum, and median values, variances, and outliers. Materials with less than 5 data points were excluded from the plot.

To further analyze the effect of material type on the final volume, we focus on pressed bales. In a setting where the current bale is pressed against the previous bale, the material type of the predecessor may have an impact on the density of the current bale. A comprehensive visualization of the impact of this previous bale material on the Length-to-Mass Ratio of the newly pressed bale (here of type HDPE) can be found in Figure 9. The subfigure on the right suggests that outliers can manifest even when the material is pressed against its same kind. In the shown case, some materials (e.g., EPS) appear to exhibit a more pronounced influence on the resulting length-to-mass ratio than others. This trend has been observed in other materials as well and highlights the challenges in achieving consistent outcomes with variable material inputs.

**Figure 10:**
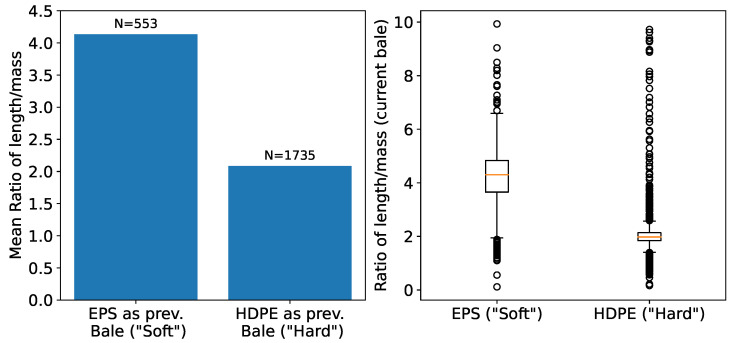
Comparative analysis of (inverse) bale density influenced by properties of previous bale materials. The left plot visualizes the impact of soft (“EPS”) and hard (“HDPE”) materials from preceding bales on the density of the current bale. The right boxplots highlight the variance in the distributions of these effects.

The impact of the previous bale material type on subsequent bale density becomes evident in Figure 10. Specifically, harder materials (e.g., HDPE) from preceding bales appear to result in more compressed bales than soft materials (e.g., foil), as indicated by the pronounced mean ratio of length to mass on the left side. However, the subfigure on the right reveals a large number of outliers within a broad range, highlighting data variability.

**Figure 11:**
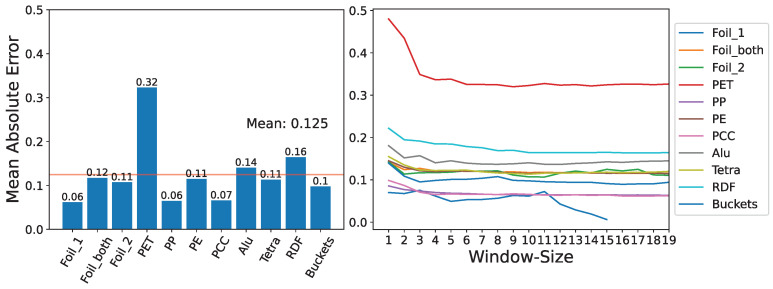
On the left, the graph depicts the MAE for predicted volume ratios (belt volume/bunker volume), calculated using the median of the last ten actual ratios for various materials at a waste sorting facility with two presses. The red line indicates the overall mean MAE. On the right, the subplot compares various median window sizes for predictions against actual ratios. Insufficient data for “Buckets” prevented testing across all time windows.

An analytical evaluation of volume ratio predictions derived from the median of the last ten actual ratios can be found in Figure 11. We observed material-specific variances in predictive accuracy, with PET manifesting the most pronounced error. When using this approach, we found that each material showed a distinct time-window size for maximal prediction fidelity. Employing the median consistently yielded superior results compared to the mean. Grouping baling events by identical bale numbers was notably less efficacious than grouping events by the same press, in a bi-press setup.

### 4.5. Inconsistent Ratio between Mass and Length

**Figure 12:**
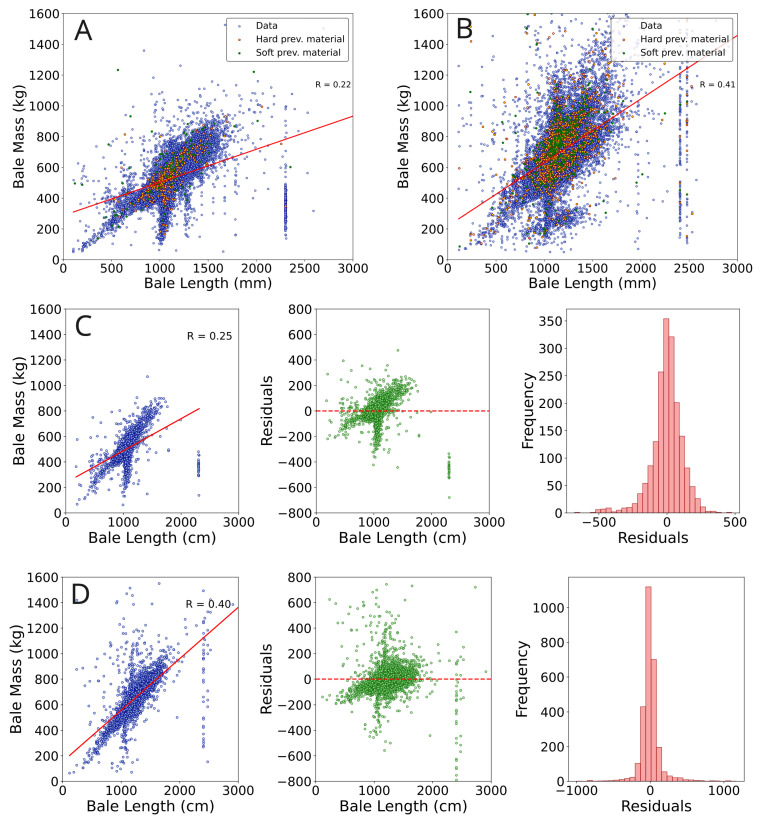
In (**A**,**B**), we present regression analyses between the measured mass (kg) and length (mm) of pressed bales from two distinct facilities, covering all materials. The quality of the previous material—categorized as “soft” or “hard”—is also indicated. For (**A**), soft materials include “Foil”, “Label-Foil”, and “Cardboard”, for (**B**), soft materials refer to “EPS”. In both cases, hard material refers to “HDPE”. We highlighted only the data where the current material differed from the previous one to emphasize the effect on different materials. (**C**,**D**) show data from these facilities for specific materials. (**C**) focuses on ’PP_White’, and (**D**) on ’PP_3D’. Both figures present (1) regression of mass vs. length for the material, (2) residual plots detailing the discrepancies between observed and predicted regression values, and (3) bar plots emphasizing the frequency distribution of residuals.

To reliably achieve a bale length near the configured optimum implies that a specific volume of a particular material type yields a predictable bale length. However, upon analyzing the ratio between the final mass and the weight of individual bales within a combination of all given material types, we have noticed deviations from the estimated regression line. This phenomenon has been consistently observed across multiple distinct facilities, as depicted in Figure 12A,B. When we examined this ratio for individual materials, we encountered a similar level of uncertainty in the data (Figure 12C,D). Reviewing the residual plots, one notices the uncertainty most prominently, as illustrated in the middle of Figure 12C,D. Considering these aspects collectively, it becomes apparent that although relevant data is generally accessible, its quality falls short in several key areas, preventing effective and meaningful control.

## 5. Machine Learning Approach

### 5.1. Volume Ratio Prediction

We perceive a predictive tool for the upcoming volume ratio $F_{1}$ as a potential solution for part of the problems at hand. Our objective was to emulate the original distribution of the actual volume ratio using a machine learning model.

We employed the Extreme Gradient Boosting (XGBoost) algorithm [22] to evaluate the significance of various predictive features across different materials. These features encompassed both present and historical data points, including current metrics such as “bunker volume” and “estimated bale count”, as well as temporal aspects like the “median of the last ten ratios” and the “time difference to the last baling of the same material”.

The preparation of our dataset involved the selection of pertinent columns to avoid data leakage, the generation of temporal additional features from the time-series data, and the exclusion of rows holding missing values. We partitioned the data into an 80% training set and a 20% testing set, following which the hyperparameters for each model were meticulously tuned through the Optuna framework [23], conducting 100 trials per model with variations in booster type, learning rate, and the number of estimators. Validation was conducted via Rolling Mean Cross-Validation with five groups, utilizing the “TimeSeriesSplit” function from the Scikit-Learn package (version 1.4.1, [24]), with mean absolute error (MAE) serving as the evaluation metric. The predictive performance was assessed by comparing the MAE between the actual volume ratio $F_{1}$ and the predictions from both the optimally tuned machine learning model and a baseline “Median_10” approach.

**Figure 13:**
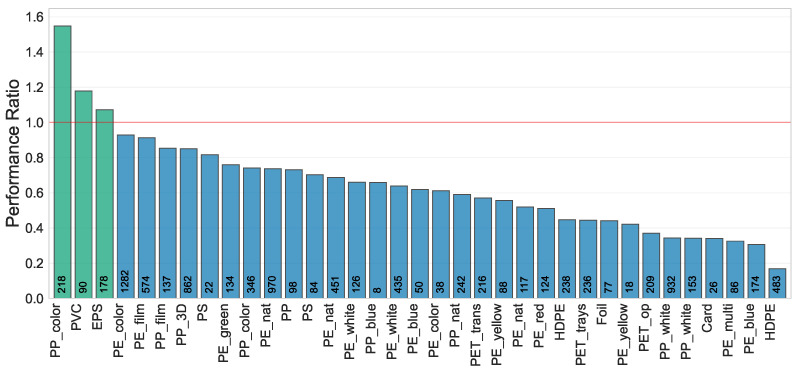
Comparison of the XGBoost model trained individually for each material against the “Median10” method in predicting the volume ratio factor, F. The performance of each model was computed with the ratio of the XGBoost model’s MAE to Median10’s MAE. A red line and color shift indicate which model had closer predictions to the actual volume ratio. The number of data points per material appears above the X-axis. The overview was generated based on the data from two waste sorting facilities.

The comparative outcomes of all per-material models developed to forecast the current ratio factor are illustrated in Figure 13. Interestingly, our predictive models scarcely outperformed the simple median of the ten most recent ratios, indicating the complexity and variability inherent in the data. The analysis revealed considerable variation in the ranking of feature importance across different materials, underscoring the volatile nature of the patterns observed.

Although neither the machine learning models nor the median approach perfectly captured the distribution of real volume ratio values, the latter consistently demonstrated superior performance in modeling accuracy compared to the XGBoost model, as detailed in Appendix A, Table A4. Subsequent testing with alternative models, including Linear Regression [25] and Random Forest Regression [26], yielded analogous results, further emphasizing the challenge of accurately predicting volume ratios in complex material handling environments.

### 5.2. Bale Length Prediction

To evaluate the inconsistency in the ratio between final bale mass and length (see Figure 12, we employed a Random Forest model [26] to predict the aggregated bale length per emptying and identify the most important features.

The model integrated various features including volume metrics (bunker and belt volumes), baling process specifics (total number of bales and total mass of bales), and time-series data (median ratio of bunker to belt volume from the ten latest measurements, and the elapsed time since the last baling process for the specific material). Before the model training phase, we conducted a data cleaning process to eliminate erroneous data and remove datasets with missing entries or insufficient size. The dataset was divided into an 80% segment for training and a 20% segment for testing without shuffling. The model’s performance was evaluated using the mean absolute error (MAE) metric, comparing the predicted bale length to the actual measured values. The Random Forest Regressor [26] yielded more accurate predictions than the XGBoost algorithm [22] in this case. The two most relevant features averaged across all models were the “total mass of bales” and the “number of bales”.

**Figure 14:**
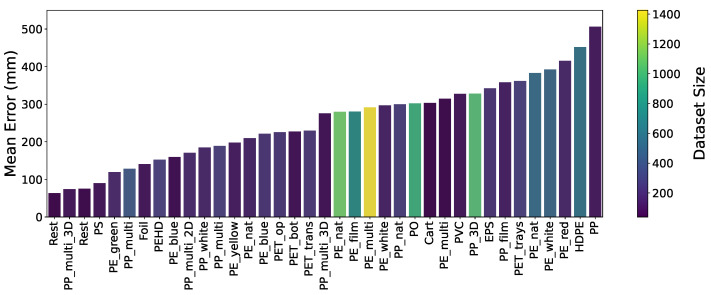
Mean prediction errors of a Random Forest model predicting the length of the total baling product, covering a range from 1 to 5 bales. The data originates from two separate waste sorting facilities, each handling different materials. The color of the bars corresponds to the dataset size.

The findings, illustrated in Figure 14, highlight the Random Forest models’ MAEs for various materials. The data aggregation pertains to baling processes that resulted in anywhere from one to five bales. It is important to note that the available data for most combinations was severely limited. The analysis revealed that the model could predict bale lengths with a mean error of approximately 24.6 cm (with a standard deviation of about 10 cm) across different materials. We further observed that the level of MAE tended to increase with an increasing number of bales. The ability of the model to predict with this insufficient level of accuracy, despite being trained on features not available at the critical decision-making point of emptying the bunker, underscores the complexity of the system under study and the need to search for material-specific solutions.

## 6. Future Work

The results of this examination underline the necessity for further exploring strategies to enhance measurement technology in waste sorting facilities.

### 6.1. Sophisticated Sensor Technology

To ensure high-quality bale output from the sorting process, an in-depth analysis of output material volumes becomes essential. Incorporating more accurate level measurements within the bunker by diversifying the sensor suite, albeit potentially higher in cost, could deliver more precise information about current material quantities. The ultrasonic sensors referenced in this study focused on the highest point, while other models may provide more comprehensive data on the 3D surface [20]. Another idea to overcome the challenge of measuring uneven surfaces may involve the installation of sensors before waste entry, above each bunker’s input conveyor belt.

When dealing with different material types, evaluating customized sensors for specific materials may be necessary. This process involves setting up potential analysis devices and confirming data from practical plants. To identify and address substantial deviations from current volume predictions, one may consider installing cameras on selected bunkers and conveyor belts. These cameras can be mapped to the data, capture critical situations, and provide valuable insights for refining the pipeline. An experimental study found that image analysis effectively determined waste composition, showing a strong correlation with physical sorting [27]. Using information about the waste composition may be a promising feature for predicting the volume and length of the bale.

### 6.2. Algorithms and Machine Learning

The current algorithm used for estimating the volume based on distance measurements from US sensors (see Appendix A, Algorithm A1) may be optimized by considering past sensor readings when detecting erroneous values, given that the belt inside the bunker did not move since and the profile of the waste remains the same. Another approach might include the application of Kalman filters for obtaining a volume estimate from several noisy sensor readings. As a recursive algorithm, Kalman filters continuously measure the state of a dynamic system, where all variables change over time [28]. This method, in conjunction with ultrasonic sensors, could potentially address the issue of noise and other uncertainties in the measurement system [29]. Key considerations for this approach include sensor accuracy, system model accuracy, initial state estimation, noise characterization, sampling rate, limited data, and potential nonlinearities in the relationship between measurements and volume.

Time series forecasting methods could also offer a valuable approach for predicting future waste volume based on historical data. Techniques such as auto-regressive integrated moving average (ARIMA) or Long Short-Term Memory (LSTM) neural networks could provide viable solutions, given their efficacy in understanding, modeling, and forecasting temporal data [30,31]. Some further conclusions about controlling the complex system of bunkers to presses may involve training an RL agent to learn the hidden dynamics of the systems and find the best points in time for emptying the bunker at an optimal volume, given the current sensory data and historical data as input. A recent study proposed an RL-based environment for regulating this specific part of a waste sorting facility [32]. To address the possible effect of data scarcity, investigating whether limited data acts as a constraint by further reducing data amounts and analyzing the resultant impact might prove insightful.

## 7. Discussion

Waste sorting facilities serve as pivotal components in the recycling industry, playing an integral role in processing and preparing waste for future use, with volume determination of waste being essential for producing standardized bales. These bales must exhibit uniformity in length to streamline both the storage and transport processes. In this context, our study delved into the viability of implementing ultrasonic sensors to measure waste volume and explored potential challenges that may arise while striving to produce bales of consistent length.

Distance sensors, including ultrasonic variants, can offer data that feed into algorithms designed for estimating waste volume. These sensors typically facilitate volume calculation and, subsequently, aid in the production of quality bales. However, our investigation revealed that several factors often undermine the efficiency of ultrasonic sensors. The findings from our research are discussed in the following.

### 7.1. Volume Ratio and Measurement Discrepancies

While ultrasonic sensors provide valuable data for volume determination, the complexities of waste pile characteristics and environmental disruptions may limit their efficiency. Our analysis reveals that reliance solely on ultrasonic sensors can yield imprecise outcomes. Factors such as varying waste material compositions, the ever-changing state of waste piles, and external noise interference play a significant role in these discrepancies. However, identifying the primary source of inaccuracies requires further investigation. The evolution of ultrasonic sensor technology should be closely monitored, as recent advancements might mitigate some of these challenges.

### 7.2. Effects and Challenges

Investigations into the factors affecting the volume ratio between bunker and belt have been conducted, with material type and the total number of bales per emptying being relevant variables. We found that a larger volume of waste, which results in a greater number of bales pressed, tends to provide a more stable and consistent volume ratio. Consequently, a strategy that emphasizes processing more bales per emptying likely enhances the predictability of these ratios.

The study revealed that volume ratios for some materials exhibited more substantial variations compared to others. The differences in spreading behavior on the conveyor belt and levels of compression that materials underwent possibly contributed to these variations. In systems where a current bale is pressed against the previous one, the material of a previously pressed bale can impact the subsequent bale’s quality and metrics. The physical properties of materials, such as their compressibility and elasticity, were proposed as potential sources of pronounced variations in quality. Recognizing the effect of these properties, especially the impact of using softer or harder materials on the compression of subsequent materials, becomes crucial when developing a machine learning model intended for bale size prediction.

Initial volume ratio estimates, based on the median of the last ten ratios, were refined through material-specific best time windows and data grouping by press type, resulting in a 12% MAE reduction for all materials together. Importantly, our findings indicate that precise bale length cannot be determined solely based on the total final mass. This raises pivotal questions regarding the potential benefits of enhancing volume estimation through the enhancement of sensors in the system. Lastly, materials such as boxes displayed significant discrepancies between their measured volume and actual density. These discrepancies, which are not captured individually, contribute to a stochastic composition of waste, representing inherent noise in the system that is currently challenging to accurately quantify.

### 7.3. Predictive Machine Learning

We see great potential in leveraging machine learning techniques, e.g., time-series forecasting, to refine volume determination in waste management. The overarching goal is to achieve higher efficiency, accuracy, and sustainability in waste management practices. However, our current efforts to employ machine learning techniques have yielded suboptimal results, underscoring the necessity of refining our approaches.

Our predictive tool for upcoming volume ratio using the XGBoost algorithm underperformed across different materials, compared to a simpler method that employs the median of the last ten volume ratios. This suggests that models capable of adaptive learning, based on shorter timeframes, may need to be explored. Similarly, a Random Forest model designed to predict bale length from various features failed to generate practical results, indicating that optimizing volume measurements alone is insufficient for accurate bale size prediction. These findings underscore the presence of missing features and temporal variations in the data, which obstruct the discernment of clear patterns by the machine learning algorithms. The fragmentation of existing datasets further compromises the reliability of these models, with scant data available for individual materials often subdivided into small segments, each associated with different material types. It necessitates the expansion and consolidation of data for each material, allowing for more effective training of predictive models.

However, variations in waste compositions and machine settings over time limit the usefulness of historical data, rendering past data sometimes less applicable to current waste volume predictions. Addressing waste management challenges requires urgent innovation, including the development of target functions and the identification of key factors such as the relationship between bale length and energy consumption in bale presses. As studies suggest, the integration of advanced machine learning techniques can significantly enhance waste sorting processes, optimize routing efficiency, and improve predictive maintenance, thereby offering substantial benefits over the long term [33].

### 7.4. Limitations

This study acknowledges several limitations that impact the generalizability and applicability of our findings. Firstly, the research was conducted under the constraint of limited data availability, due to changes in machine settings and database. Secondly, due to the operational constraints within the waste sorting facilities, there was no opportunity to modify existing technological setups or introduce innovations (e.g., LIDAR sensors) that could potentially enhance the accuracy of volume determination by providing a full height profile. The models we developed were trained based on the configurations of three European waste sorting facilities, suggesting our findings are most applicable to facilities with similar setups. Facilities with different configurations may necessitate the development and training of new models to achieve comparable findings. Future research efforts are encouraged to address these constraints.

## 8. Conclusions

This study revealed marked discrepancies in volume measurements between storage bunkers and conveyor belts, underscoring the need for a multi-faceted approach to waste volume determination to enhance the production of standardized, quality bales. Although ultrasonic sensors provide valuable insights, they cannot be solely relied upon due to the inherent challenges associated with varying waste compositions, the unstable state of waste piles, external interference, and variations in material-specific densities.

Multiple aspects make this setting a challenging field for Machine Learning. The reliability of predictions is hampered by limited datasets, undocumented changes in machine configurations, and sensor malfunctions. Our findings imply that poor data quality caused by sensory limitations cannot be compensated by processing the data with current machine learning methods. Major improvements are anticipated from two distinct areas, enhancing the system of sensors and developing adequate algorithms to efficiently handle the sensory data and make adequate predictions.

Testing some of the proposed approaches may allow for a more accurate waste volume determination, promoting sustainability that is beneficial to both the environment and society.

## Data Availability

In compliance with confidentiality agreements, the raw data used in this study are not publicly available. However, we are committed to promoting transparency and reproducibility in research. The data processing scripts used in this study are available upon request to the first author.

## Appendix A

**Algorithm A1** Calculating Bunker Volume from Sensor Data
1: **procedure** BunkerVolume( sensorValuesList,mountHeightList,bunkerWidth,bunkerLength ) 2: numSensors←lengthofsensorValuesList 3: numValidSensors←0 4: avgHeight←0 5: **for** ctr,(sensorValue,mountHeight) in enumerate(zip(sensorValuesList,mountHeightList)) **do** 6: **if** ctr=0 **then** 7: sensorValuePrev←−1 8: mountHeightPrev←0 9: sensorValueNext←sensorValuesList[ctr+1] 10: mountHeightNext←mountHeightList[ctr+1] 11: **else if** ctr=numSensors−1 **then** 12: sensorValuePrev←sensorValuesList[ctr−1] 13: mountHeightPrev←mountHeightList[ctr−1] 14: sensorValueNext←−1 15: mountHeightNext←0 16: **else** 17: sensorValuePrev←sensorValuesList[ctr−1] 18: mountHeightPrev←mountHeightList[ctr−1] 19: sensorValueNext←sensorValuesList[ctr+1] 20: mountHeightNext←mountHeightList[ctr+1] 21: **end if** 22: **if** 0<sensorValue<mountHeight **then** 23: avgHeight←avgHeight+mountHeight−sensorValue 24: numValidSensors←numValidSensors+1 25: **else if** (0<sensorValuePrev<mountHeightPrev) and (0<sensorValueNext<mountHeightNext) **then** 26: sensorValueTemp←(sensorValuePrev+sensorValueNext)/2 27: mountHeightTemp←(mountHeightPrev+mountHeightNext)/2 28: avgHeight←avgHeight+mountHeightTemp−sensorValueTemp 29: numValidSensors←numValidSensors+1 30: **else if** 0<sensorValuePrev<mountHeightPrev **then** 31: avgHeight←avgHeight+mountHeightPrev−sensorValuePrev 32: numValidSensors←numValidSensors+1 33: **else if** 0<sensorValueNext<mountHeightNext **then** 34: avgHeight←avgHeight+mountHeightNext−sensorValueNext 35: numValidSensors←numValidSensors+1 36: **end if** 37: **end for** 38: **if** numValidSensors>0 **then** 39: avgHeight←avgHeight/numValidSensors 40: volume←avgHeight*bunkerWidth*bunkerLength 41: **else** 42: volume←0 43: **end if** 44: **return** volume 45: **end procedure**

**Table A1:** List of abbreviations related to parameters and measurements used in the waste sorting process. Each abbreviation is followed by its description and examples. These parameters serve as the foundation for several figures presented in this document.

Abbreviations	Description	Examples
_unix_dt	Unix timestamp in date format	1628000000, 1635001200, *…*
_dt	Date	2023-07-31, 2023-08-01, *…*
TargetName	Target name	Press_1, *…*
BaleID	Bale ID	001, 002, 003, *…*
Impulse	Impulse	25, 30, *…*
BunkerName	Bunker name	Bunker A, Bunker B, *…*
BunkerVolume	Bunker volume right before emptying (US)	17, *…*
BeltVolume	Belt volume of full flow batch (laser)	15, *…*
RecipeNumber	Recipe number	3, 4, *…*
MaterialNumber	Material number	001, 002, 003, *…*
MaterialName	Material name	PET, PE, *…*
CorrelationFactor	BunkerVolume/BeltVolume	0.95, 0.98, 1.00, *…*
ImpulseVolume	Impulse volume	0.7, *…*
Regular	Regular	−1, 1
External	External	−1, 1
BaleVolumeTarget	Bale volume (target)	9.5, *…* m^3^
BaleVolumeCalculated	Bunkervolume/Number of produced bales	10.5, *…* m^3^
BaleVolumeActual	BaleVolumeCalculated × CorrelationFactor	8.5, *…* m^3^
ActiveEnergy	Active energy	2850, *…*
ReactiveEnergy	Reactive energy	0, *…*
ApparentEnergy	Apparent energy	0, *…*
BindingImpulses	Binding impulses	5, 6, 7, *…*
ActualLength	Actual length	1205, *…*
ActualMass	Actual mass	625, *…*
TargetImpulsesConfig	Target impulses configuration	Company’s requirements
TargetImpulses	Target impulses	Company’s requirements
TargetLength	Target length	Company’s requirements
TargetMass	Target mass	Company’s requirements

**Table A2:** Abbreviations and Descriptions from Ultrasonic Sensors.

Abbreviations	Description	Examples
_unix_dt	Unix timestamp in date format	1628000000, 1635001200, *…*
_dt	Date and time	2023-08-01 12:30:00, 2023-08-01 12:35:00, *…*
h_US_0	Ultrasonic sensor 0 height measurement	865, 1783, *…* [mm]
h_US_1	Ultrasonic sensor 1 height measurement	865, 1783, *…* [mm]
h_US_2	Ultrasonic sensor 2 height measurement	865, 1783, *…* [mm]
h_US_3	Ultrasonic sensor 3 height measurement	865, 1783, *…* [mm]
h_US_4	Ultrasonic sensor 4 height measurement	865, 1783, *…* [mm]
h_US_5	Ultrasonic sensor 5 height measurement	865, 1783, *…* [mm]
MountingHeight_US_0	Mounting height of sensor 0	1953, 2035, *…* [mm]
MountingHeight_US_1	Mounting height of sensor 1	1953, 2035, *…* [mm]
MountingHeight_US_2	Mounting height of sensor 2	1953, 2035, *…* [mm]
MountingHeight_US_3	Mounting height of sensor 3	1953, 2035, *…* [mm]
MountingHeight_US_4	Mounting height of sensor 4	1953, 2035, *…* [mm]
MountingHeight_US_5	Mounting height of sensor 5	1953, 2035, *…* [mm]
Volume	Volume measurement	6.5, *…*
Active	Activity status	Yes, No
Read_Door_Left_Open	Read status of left door (open)	0, 1
Read_Door_Left_Closed	Read status of left door (closed)	0, 1
Read_Door_Right_Open	Read status of right door (open)	0, 1
Read_Door_Right_Closed	Read status of right door (closed)	0, 1
Read_Bunker_Belt_Left	Read status of left bunker band	0, 1
Read_Bunker_Belt_Right	Read status of right bunker band	0, 1

**Table A3:** This table provides the material abbreviations, their corresponding full descriptions (Description), and examples for each material. These materials are processed in the examined waste sorting facilities and form the basis for all other figures in this document.

Abbreviations	Description	Examples
Card	Cardboard	Boxes, packaging materials
Cardboard	Cardboard Packaging	Cardboard boxes, food packaging
Cart	Cartridges	Cartridges for printing, etc.
Cont	Contaminants	Non-recyclable materials mixed in with the recyclables
EPS	Expanded Polystyrene (Styrofoam)	Coffee cups, packaging peanuts
Foil	Foil	foil packaging
HDPE	High Density Polyethylene	Sturdy containers, corrosion-resistant piping
Label_Foil	Label Foil	Foil used in labels, often with an adhesive on one side
PCC	Paper Cardboard Carton	Copy paper, boxes, etc.
PE	Polyethylene	Plastic bags, plastic films, containers
PE_blue	Polyethylene Blue	Blue plastic containers
PE_color	Polyethylene Colored	Colored plastic containers
PE_color	Polyethylene Colored	Colored plastic containers
PE_film	Polyethylene Film Type 1	Shrink wrap, plastic food wrap
PE_green	Polyethylene Green	Green plastic containers
PE_multi	Polyethylene Multi-Colored	Multi-colored plastic products
PE_nat	Polyethylene Natural	Plastic bags, bottles
PE_red	Polyethylene Red	Red plastic containers
PE_white	Polyethylene White	White plastic containers
PE_yellow	Polyethylene Yellow	Yellow plastic containers
PET_bot	Polyethylene Terephthalate Bottles	Beverage bottles, like water or soda bottles
PET_op	Polyethylene Terephthalate Opaque Bottles	Certain types of beverage bottles
PET_trans	Polyethylene Terephthalate Clear Bottles	Water bottles
PET_brown	Polyethylene Terephthalate Brown	Brown PET products, like bottles
PET_clear	Polyethylene Terephthalate Clear	Clear PET products, like beverage bottles
PET_green	Polyethylene Terephthalate Green	Green PET products, like bottles
PET_multi	Polyethylene Terephthalate Multi-Colored	Multi-colored PET products
PET_opaq	Polyethylene Terephthalate Opaque	Non-transparent PET products
PET_tray	Polyethylene Terephthalate Tray	Trays made from PET, often for food packaging
PET_trays	Polyethylene Terephthalate Trays	Food packaging trays
PO	Polyolefin	Various types of packaging films
PP	Polypropylene	Bottle caps, yogurt containers, toys
PP_3D	Polypropylene 3D	3D printed objects
PP_blue	Polypropylene Blue	Blue plastic containers made from polypropylene
PP_color	Polypropylene Colored	Colored polypropylene products
PP_color_2D	Polypropylene Colored 2D	Colored polypropylene products
PP_color_3D	Polypropylene Colored 3D	Colored polypropylene products
PP_film	Polypropylene Film	Snack packaging
PP_green	Polypropylene Green	Green plastic containers made from polypropylene
PP_nat	Polypropylene Natural	Non-dyed polypropylene products
PP_red	Polypropylene Red	Red plastic containers made from polypropylene
PP_white	Polypropylene White	White plastic containers made from polypropylene
PP_yellow	Polypropylene Yellow	Yellow plastic containers made from polypropylene
PS	Polystyrene	Disposable cutlery, CD cases
PVC	Polyvinyl Chloride	PVC pipes, vinyl flooring
Rest	Residual Waste	Waste that cannot be recycled or composted
Tetra	Tetra Pak	carton packaging for beverages

**Table A4:** Results of the volume ratio prediction (5.1), comparing the mean absolute error (MAE) between the bunker–belt volume ratio $F_{1}$ and the predictions of both an XGBoost-model (“MAE (model)”) and the median of its last ten values (“MAE (median 10)”). The performance ratio is calculated by dividing the column “MAE (median 10)” by “MAE (model)”.

Material	Datapoints (n)	MAE (Median 10)	MAE (Model)	Performance Ratio
PP_color	218	0.1135	0.0734	154.72
PVC	90	0.127	0.1078	117.86
EPS	178	0.1184	0.1105	107.16
PE_film_2	562	1.1075	1.1165	99.19
PE_color	1282	0.0859	0.0925	92.92
PE_film_1	574	0.0387	0.0424	91.34
PP_film	137	0.0837	0.098	85.35
PP_3D	862	0.1387	0.163	85.06
PS	22	0.1221	0.1497	81.56
PE_green	134	0.077	0.1015	75.89
PP_color	346	0.089	0.12	74.15
PE_nat	970	0.0861	0.117	73.65
PP	98	0.074	0.1012	73.12
PS	84	0.0884	0.1258	70.26
PE_nat	451	0.0686	0.0997	68.75
PE_white	126	0.108	0.1637	65.98
PP_blue	8	0.1332	0.2023	65.82
PE_white	435	0.0526	0.0825	63.81
PE_blue	50	0.0864	0.1395	61.91
PE_color	38	0.132	0.2157	61.17
PP_nat	242	0.073	0.1237	59.01
PET_trans	216	0.0805	0.1409	57.11
PE_yellow	88	0.0834	0.1499	55.65
PE_nat	117	0.0784	0.1508	51.96
PE_red	124	0.0951	0.186	51.15
HDPE	238	0.0733	0.164	44.70
PET_trays	236	0.0771	0.1737	44.39
Foil	77	0.0841	0.1909	44.07
PE_yellow	18	0.102	0.2422	42.11
PET_op	209	0.0694	0.1872	37.09
PP_white	932	0.0452	0.1316	34.35
PP_white	153	0.0655	0.1918	34.17
Card	26	0.1174	0.3452	34.01
PE_multi	86	0.0416	0.1278	32.56
PE_blue	174	0.0685	0.2242	30.57
HDPE	483	0.0927	0.5479	16.91
